# A qualitative analysis of perceptions of various stakeholders on nutrition-sensitive agricultural interventions, including the taxation on sugar-sweetened beverages (SSBs), to improve overall health and nutrition in South Africa

**DOI:** 10.1186/s12889-020-09440-8

**Published:** 2020-09-03

**Authors:** Tayla Ashton Kaltenbrun, Lisanne Monica du Plessis, Scott Drimie

**Affiliations:** Division of Human Nutrition, Department of Global Health, Faculty of Medicine and Health Sciences, Stellenbosch University, PO Box 241, Cape Town, 8000 South Africa

**Keywords:** Sugar-sweetened beverages, Double burden of malnutrition, Small-scale farmers, Nutrition-sensitive agricultural policies

## Abstract

**Background:**

As a low-middle income country, South Africa has seen an upsurge in the double burden of malnutrition (DBM). Owing to the rising costs of obesity on healthcare in South Africa, the National Treasury implemented a fiscal policy for the taxation of SSBs, known as the Health Promotion Levy, in line with the WHO recommendation. Potential negative impacts of the policy on the sugar cane industry and economic and rural development have been voiced by different sectors. By including a subsection in the SSBs fiscal policy and aligning the goals with existing policies, government could have made provisions for sugar cane farms to substitute crops with alternatives, including nutritional alternatives where possible, while supporting existing small-scale farms to produce nutrient-dense, local and culturally acceptable crops. Thus, the purpose of the study is to understand the perceptions of the various stakeholders on combining nutrition-sensitive agricultural interventions with the taxation on sugar-sweetened beverages (SSBs) to improve overall health and nutrition in South Africa.

**Methods:**

Semi-structured, in-depth interviews were conducted with each participant. The interviews were audio-recorded, transcribed intelligent verbatim, and cross-checked against the audio-recordings by the principal researcher. ATLAS.ti 8 software was used to navigate the data and assist with thematic analysis.

**Results:**

Perceptions of combining SSB taxation with agricultural policies to improve food and nutrition security were positive. The participants found it to be an innovative idea in theory but questioned the feasibility of combining policies. Participants highlighted education as an essential element for successfully changing behaviour to ensure a positive impact of the combined policy approach. Participants believed that before government could scale up nutrition-sensitive agricultural interventions, basic services and government functions would first need to run optimally.

**Conclusion:**

Overall, perceptions with regard to combining the taxation on SSBs with nutrition-sensitive agricultural policies to improve overall health and nutrition in South Africa were positive. Although participants questioned the feasibility of combining these policies, it was viewed as a way to combat alleged collateral damage linked to the tax, with a specific focus on developing small-scale farmers. More research into these combined policy approaches in a South African context is required.

## Background

As a low-middle income country, South Africa has seen an upsurge in the double burden of malnutrition (DBM). According to the South Africa Demographic and Health Survey (SADHS), the prevalence of hypertension, overweight and obesity have increased since 1998 [1, 2]. In the context of the rising prevalence of obesity and obesity-related diseases, such as type II diabetes mellitus and cardiovascular disease, South Africa still faces public health concerns in many communities in the form of undernutrition and household food insecurity [1, 2]. Due to the rising consumption of sugar-sweetened beverages (SSBs) and the possible contribution to the obesity epidemic, the World Health Organization (WHO) recommended that countries include a fiscal policy to reduce the consumption of SSBs [2].

The prevalence of obesity in South Africa is one of the highest in sub-Saharan Africa, while it still bears the burdens of stunting and hidden hunger [1]. Owing to the rising costs of obesity on healthcare in South Africa, the National Treasury implemented a fiscal policy for the taxation of SSBs, known as the Health Promotion Levy, in line with the WHO recommendation [3]. The WHO reports that a 20% tax on SSBs is required to reduce the purchasing and consumption of SSBs and have an impact on health [4]. The tax, which excludes 100% fruit juice, was intended to be set at a tax rate of 2,21 cents per gram of sugar over the 4 g per 100 ml threshold, per beverage [4, 5].

However, it currently only roughly equates to an 11% tax incidence, which may only have a marginal impact on consumption and health-related outcomes [4, 5]. This policy, introduced by the Department of Health and implemented by the National Treasury, is seen as a cost-effective measure to combat the obesity crisis and reduce the consumption of SSBs nationwide, offsetting the future burden on the health system and broader economy, while generating revenue for government which potentially could be put back into healthcare [3].

Currently, there is no link between the tax and health allocations in the budget. Ring-fencing of any tax is against the National Treasury’s policies, and any money generated goes into the National Revenue Fund for general government spending. Potential negative impacts of the policy on the sugar cane industry and economic and rural development have been voiced by different sectors [6]. Prospective research illustrating the impact of the tax on reducing obesity and non-communicable diseases (NCDs) is yet to be published; however, a modelling study by Manyema et al. [7] predicted that a 20% tax on SSBs will reduce obesity by 2,4% and 3,8% in females and males, respectively, using price elasticities.

Agriculture for nutrition policies have been proposed as a mechanism to improve food security and positively impact a country’s health and economic outcomes [6, 8, 9]. Such policy and programme approaches are termed “double” or “triple gains”^1^ by the WHO and assist in combating the DBM [10].

In South Africa, people with lower socioeconomic status have inadequate availability and access to balanced, nutritious diets. Most of these people rely on social grants and fail to achieve dietary diversity through the type of food afforded and consumed. This contributes to the DBM seen in many households in rural areas. Hidden hunger, caused by insufficient intake of vitamins such as vitamin A, zinc and iron, is a common problem in these households. A systematic review by Misselhorn and Hendriks [11] explored the commonly highlighted causes of food insecurity in South Africa. Poverty and lack of income were most frequently reported as the main drivers of food insecurity [11, 12].

According to Statistics South Africa (Stats SA), 26% of households have inadequate access to food. High levels of unemployment and poor purchasing power result in the increased consumption of more affordable processed foods together with staple grains, which tend to be higher in energy with poor nutritional value [11, 12]. Other highlighted causes were the lack of agricultural inputs and travelling distance from food markets [12]. By including agriculture for nutrition in the fiscal policy paper, the agricultural sector can work with education, infrastructure development and departments for social protection to improve nutrition [11, 13–15].

There is no literature which investigates the possibility of combining the taxation of SSBs with a nutrition-sensitive agricultural policy in South Africa. This study explores the potential for including a subsection on agriculture for nutrition in the fiscal policy paper, to offset the negative ramifications of the fiscal policy for rural and economic development and any potential job loss within the sugar cane industry.

This study investigated the feasibility of utilising the revenue generated from the tax to drive rural development by supporting sugar cane farms to substitute crops, with nutritional alternatives where possible, while supporting existing small-scale farms to produce nutrient-dense, local and culturally acceptable crops. The study also explored the likely benefits of combining nutrition-sensitive and nutrition-specific policy approaches for food and nutrition security. This potential inclusion of agriculture in the policy would need to be combined with well-designed outcomes for monitoring and evaluation. This would allow investors to see the combined impact of tax on SSBs and subsidised nutritious crops on food and nutrition security as well as malnutrition [6, 8, 9].

## Methods

This study used semi-structured, in-depth interviews (*n* = 16) to obtain data. In-depth interviews allowed adequate exploration of the perceptions of various stakeholders on combining nutrition-sensitive agricultural interventions with the taxation on SSBs. During the interviews, the principal researcher asked a series of open-ended questions to obtain a detailed and comprehensive understanding of the topics.

In qualitative research, there is no strict guideline for calculating sample size and study power. Therefore, the concept of data saturation was used in this research to guide the conclusion of data collection. The researcher continued to sample until no new data about the phenomena was collected.

### Sample

The study frame included the following five sectors in Gauteng, South Africa (restricted to Gauteng because of technical and logistical convenience): the health sector, the food and beverage industry sector, the agricultural sector, the finance sector and the consumer interest sector. An interview could not be secured with an association related to the sugar industry. Therefore, reference is made to their official position statement on the Health Promotion Levy on sugary beverages. These sectors were identified as key stakeholders for combining nutrition-sensitive agricultural interventions with the taxation on SSBs. The study population included subject matter experts, managers and senior managers or personnel with knowledge and experience regarding the phenomena under study. Intensive, purposive sampling of one to three participants from each of the above-mentioned sectors was done to identify suitable participants. Once the interviews commenced, snowball sampling was used to help identify other appropriate interview candidates.

The final sample size consisted of three participants from the health sector, three participants from the food and beverage industry sector, six participants from the agricultural sector, two participants from the finance sector and two participants from the consumer interest sector (*n* = 16).

### Data collection

Semi-structured, in-depth interviews were conducted with each participant to collect data. Each interview was conducted in English and lasted between 40 and 90 min. Individuals were contacted via telephone and email to give them a breakdown of the study and request their participation. Each interview was conducted once in person, as a face-to-face interview, and took place at the participants’ convenience.

The principal researcher used an interview guide, which was developed for the purpose of this study (Additional file 1). The guide consisted of a series of key, pre-identified themes with accompanying questions and prompts, to allow for open discussions during each interview, while ensuring all key research questions were covered. The key, pre-identified themes covered in each interview were: understanding and perceptions of the topic areas, food and nutrition security, DBM, economic environment, enabling environment, scaling up nutrition-sensitive agricultural policies and recommendations.

All participants received written information explaining the study and signed individual consent forms. Ethics approval for the interviews was granted by the Health Research Ethics Committee of the University of Stellenbosch (Ref. no. S18/10/212) and the National and Provincial Health Research and Ethics Committee of South Africa (Ref. no. GP_201901_034). Confidentiality was ensured by not recording any personal identification data.

### Data analysis

The semi-structured, in-depth interviews were audio-recorded (with permission), transcribed intelligent verbatim, and cross-checked against the audio-recordings by the principal researcher. The ATLAS.ti 8 software was used to navigate the data and assist with thematic analysis. After familiarisation with the raw data, interview transcripts were imported to ATLAS.ti 8 and coded using pre-set themes and emergent themes. Codes were grouped according to relevant themes and a spider diagram was created by cross-checking the themes and codes against the transcripts and the literature review. The network function on ATLAS.ti 8 was used to recreate spider diagrams on the main themes and look for further patterns, connections and relationships between and within themes (Additional file 2). Data on code groups and quotations were exported onto a Microsoft Word document and documented in the results section.

## Results

The in-depth interviews provided insights into the perceptions of various stakeholders on the current state of food and nutrition in South Africa, along with their opinions regarding the current taxation on SSBs and the feasibility of combining the fiscal policy with nutrition-sensitive agricultural interventions.

Of the sixteen participants interviewed, half were women. The participants’ ages ranged from 30 to 74 years. Postgraduate qualifications were held by ten of the participants, and all participants had more than 5 years’ work experience within their relevant field or sector. The key findings identified within each theme are discussed below.

### Food and nutrition security

The study participants described South Africa to be a food secure nation when considering the availability of food nationally. However, they highlighted problems relating to household food security, especially in terms of accessibility and affordability of nutritious foods. This was voiced as an ongoing concern for consumers with lower socioeconomic status. These factors were linked to the current concern about the DBM and the NCDs epidemic in South Africa.

I think on a national level South Africa is food secure; at a household level that is not always the case. I think there is massive disparity in our communities, with ‘those who didn’t have’ … becoming ‘those who have’ but maybe not making healthy choices.

(Agricultural sector)

Participants reported that government currently engages in adequate programmes to assist in improving food security; however, those from the health and agricultural sector thought that the overall intended outcome of some of these programmes are not being realised.

That’s the way the allocation works, so, ultimately it depends on political will if you want, if someone is going to allocate to nutrition-specific or nutrition-sensitive interventions.

(Finance sector)

Participants from the agricultural and finance sector expressed the importance of focusing on stability as an essential pillar of food security. They raised concerns about food production under current environmental conditions, such as climate change and population growth. They also stressed the need to adopt efficient agricultural practices to cope with these pressures, as well as to review legislation restriction regarding food waste.

Participants from the agriculture, health and food and beverage industry sector also queried the ability of sugar cane farmers to switch over to a different crop production, especially fruits and vegetables. Reasons provided are the different agricultural systems, relative difficulty, past training, inadequate knowledge and climate. Suggestions for sugar cane farmers were to utilise the revenue generated to improve upon rotational crops, produce sugar cane for biofuels and/or produce alternative sugars or sweeteners such as xylitol or stevia.

Combining the policies was seen as a positive way of overcoming the alleged collateral damage linked to the tax, with a specific focus on developing small-scale farmers, creating jobs and ensuring adequate household food security in rural populations.

So, I suppose if you consider it from a development perspective, the funds would have to be applied to small-scale and subsistence farmers – to enable them to increase their yields and participate in markets.

(Agriculture sector)

Participants from the agriculture, health and food and beverage industry sector also seemed to agree with utilising the funds to subsidise healthier foods or provide food vouchers, thus making the healthier choice the easier choice. When asked where they thought the revenue generated from the tax should go to improve overall food and nutrition security, participants highlighted youth development and mass media education. Participants consistently mentioned education as an essential element required to successfully change behaviour and ensure a positive and sustainable consumer impact of the taxation on sugar and other combined policy approaches.

There needs to be more done to educate consumers in terms of making healthy lifestyle choices, with respect to total nutrition. If the consumer is not educated enough to make the right decisions and the right choices, no initiatives that were put in place are actually going to work.

(Food and beverage industry sector)

### Sugar tax, negative externalities and education

In general, participants perceived the taxation on SSBs to be a solitary measure implemented by government to combat the obesity epidemic in line with global trends. The participants, except those from the health sector, expressed concerns over the true motivation for the tax, as it is seen more as a revenue-generating policy than a health-related policy. This is mainly due to the lack of ring-fencing of the funds for health-related initiatives such as health promotion.

The tax is not a silver bullet; it is one important intervention that needs to be part of a broader package of interventions.

(Health sector)

We think it was also misguided, but the biggest problem was that it was introduced without any associated programmes – to either publicise or monitor what is happening in the marketplace.

(Consumer interest sector)

Participants from the financial and agricultural sector agreed that funds are generally not ring-fenced from fiscal policies for certain programmes. However, participants from the health sector responded that the revenue generated would allow government to allocate a greater share of funds from the general fiscus to the health budget.

The health sector and government participants from the agricultural sector were more in favour of the tax, seeing it as a step in the right direction. They perceived that it will have a positive impact on consumers and will likely produce the intended health benefits. Although, in general, participants voiced concerns about possible negative externalities which could arise due to the tax, namely the negative impacts on the food and beverage, sugar cane and small-scale farming sectors – thus impeding economic growth and rural development on top of the existing political, social and economic challenges in South Africa.

From a health point of view, it is good; from an agricultural point of view it is good and bad.

(Agricultural sector)

For the health issues, it will be okay, but if you look at it from a social impact perspective, it will definitely have a negative impact; but there will be some positive health benefits gained from the tax.

(Health sector)

It appeared that all the participants except those from the health sector thought that the tax would be relatively ineffective, only having a marginal impact on consumers. This was related to largely unknown price elasticities and sensitivities in South Africa, the relative proportion of household budget spent on SSBs, the statistical inflation implication of the tax for 1 year and an inability to change consumer behaviour without other awareness or education measures.

Participants from the agriculture and health sector agreed that funds generated from the taxation on SSBs should be directed back into healthcare, with a focus on areas such as youth development and mass media education, for the proposed benefits regarding obesity and NCDs to be realised. Whereas participants from the food and beverage industry sector identified that the revenue generated should be directed towards subsidising healthier food options.

It should be noted that participants from the health sector highlighted that the tax is still relatively new, thus impacts on consumption, obesity and job losses would have to be assessed over a longer time frame. Also, any negative impact may be outweighed by the overall positive health benefits.

I think the tax on SSBs is still at its infant stage. With time, the benefits will come from the Health Promotions Levy and the awareness … we have already started to see the awareness. In Pretoria, you can see they have started putting up the billboards for this.

(Health sector)

Participants mentioned that obesity and NCDs are highly complex and would need to be tackled by utilising a holistic, multidimensional and multisectoral approach. Participants also emphasised the substitution effect, whereby consumers would likely switch to another beverage or food product that may not necessarily be healthier.

I don’t think a tax like that builds the right behaviours in people, because people can switch from sugar in a soft drink because it is expensive, they can get excess amount of sugar from somewhere else. So, for me if there was education we would be on a better route, than just implementing the tax.

(Food and beverage industry sector)

Moreover, participants attributed likely health benefits to industry adaptation of SSBs rather than reduced consumption.

### Double burden of malnutrition

The DBM was perceived to be both a public health and an economic burden, with a growing negative impact on social and youth development. Participants mentioned that it places a greater strain on the already overburdened public health sector, which does not have the capacity to deal with the current NCDs epidemic.

Participants from the finance, consumer interest and food and beverage industry sector perceived that the sugar tax was adopted as a last attempt to curb the obesity epidemic, which rather requires a package of interventions. It was also continuously referred to as a revenue-generating tax for a government which is approaching a fiscal cliff unless government spending is cut.

That is treating the symptom and not the cause; the cause is at ground level.

(Food and beverage industry sector)

Participants from the agriculture, health and food and beverage industry sector thought that combining nutrition-sensitive with nutrition-specific policies would assist in improving the DBM.

They would, but it also goes with education; if it is not coupled with education and awareness making, capacity building, then it is useless – it is within a community, making certain informed choices.

(Agriculture sector)

The consumer interest sector also mentioned that having an independent, government-facilitated consumer movement group in South Africa is essential.

### Government function and scaling up nutrition-sensitive agricultural interventions

When asked if the tax on SSBs could be combined with a nutrition-sensitive agricultural policy, participants highlighted that before government can develop and implement such policies, it needs to ensure that basic services are scaled up and run optimally.

So, I mean from a government perspective they need to ensure that the public services that they provide are working optimally. So, good roads, constant electricity and things like that are basic things that we are not even covering at the moment.

(Agriculture sector)

Moreover, participants mentioned that once basic services are functioning optimally, a demand needs to be created for healthy foods within a market-based economy. If there is no demand for healthy products, it will be difficult to make these products available, accessible and affordable.

Essentially, functioning in a market-based economy – the incentive is the return you are going to get on your product, so if you want to incentivise nutritious production of goods there needs to be an attractive price or an attractive market. I mean, you could subsidise it, but you still haven’t created a demand for that product.

(Agriculture sector)

The participants, except those from the consumer interest sector and some from the agricultural sector, were in favour of scaling up nutrition-sensitive agricultural interventions, as long as government effectively carries out its main functions before embarking on extended welfare policies and interventions.

Thereafter, government can focus on not only agriculture but the consumer, concentrating on education, community engagement and empowerment. The participants agreed that South Africa has some of the best policies in the world; however, there is a need for greater policy coherence and stakeholder involvement to improve implementation, monitoring and evaluation.

The policy and operational weaknesses identified by the participants, which could hinder scaling up such interventions in future, were poor coordination, overlap, animosity, lack of responsibility, poor monitoring and evaluation, inadequate impact analysis, poor stakeholder involvement, lack of capacity, funding and government departments working in silos.

They [government] simply do not have the capacity or the infrastructure. Budget, knowledge, experience and capacity. Those are the stumbling blocks.

(Consumer interest sector)

To monitor and evaluate the effectiveness and efficiency of agriculture for nutrition policies, participants mentioned different indicators. These indicators could be grouped to form a composite of indicators that assess the impact of these policies on health and nutrition across the value chain. The indicators mentioned are production indicators, purchasing patterns, consumption and dietary diversity patterns and health status indicators.

You would need to have very strict monitoring and evaluation mechanisms in place. You need to get people on board, not just at national level but also you need to filter down.

(Health sector)

### Collaboration and enabling environments

Participants mentioned that collaboration between departments and sectors would need to be enforced or combined with a performance indicator, thus incentivising different departments to work together and ensure greater accountability and responsibility. Participants also said that combined policy approaches would require clear, balanced, strategic goals and greater stakeholder buy-in from policy development to monitoring, evaluation and policy review. This would ensure less lobbying and push-back from different sectors.

Nutrition champions were highlighted as essential role players in scaling up nutrition-related initiatives, collaboration for policies and creating enabling environments.

### Economic environment

When asked if combining nutrition-sensitive and nutrition-specific policies would have an impact on the South African economy, participants perceived that it would have an indirect positive impact. This is due to job creation opportunities across the value chain, together with having a healthier, more productive society and greater youth development opportunities.

So, if it results in increased production and stimulates a more effective and efficient production it will be positive for the gross domestic product and for our economy.

(Finance sector)

I think it is extremely difficult to make these things work. I think unless you manage their performance based on certain things, unless the monitoring and evaluation frameworks requires them to explicitly work together, you are not going to see cooperation.

(Finance sector)

## Discussion

### Food and nutrition security

With regard to food and nutrition security, participants were more concerned with the accessibility and affordability pillars of food security, as opposed to the availability of food. These barriers were linked to the growing prevalence of overweight and obesity and NCDs in South Africa. These concerns were found to be consistent with research conducted in South Africa which also highlights poverty and lack of income as common causes of food insecurity within an obesogenic environment [11, 12, 16].

Participants stressed the need to focus on sustainable agricultural practices in line with climate change actions to ensure greater food security for the future. Thus, the ongoing research on biofortified crops that are water efficient and resistant to abiotic stressors could be combined with the development of agriculture for nutrition policies. This would allow South Africa, as a water-stressed country, to potentially benefit from sustainable alternative crops which reduce water usage [11–13].

Concerning the participants’ apprehension on governments ability to combine policies due to various barriers, it is important to note that stand-alone policies have been found to have a relatively limited impact on food and nutrition security [17–19]. A study which investigated drivers for political commitment to nutrition found that low-income countries frequently reported that nutrition and international actor networks, civil society mobilisation, vertical coordination and capacities and resources are essential for driving government commitment for nutrition [20]. These key components for combined policy success are ways in which the mentioned barriers, such as political will, competing priorities and legislation restrictions, could be overcome.

Participants mentioned that sugar cane farmers would have difficulty in switching from their current harvesting practices, to growing fruits and vegetables. To overcome this, participants mentioned utilising the revenue generated from the tax to improve upon rotational crop production in line with nutritious foods. Crop rotation is not commonly practised in the South African sugar cane industry, although it has been shown to benefit maize and wheat crops [21]. A study conducted in KwaZulu-Natal, South Africa, found that soybean and sugar cane crop rotation was beneficial for cane production [22]. Crop rotation has the potential to provide nutritious food to small-scale farmers and surrounding communities, thus combating the potential negative externalities that could arise from the tax.

Participants also recommended that sugar can farmers engage in the biofuel industry as a way of combating the negative externalities and improving livelihoods. However, studies have found that greater government support is needed for sugar cane to be produced for biofuels. Government support will assist in ensuring the viability of biofuels in current markets, as highlighted in the official position statement from the sugar industry [23].

Overall, perceptions with regard to combining nutrition-specific and nutrition-sensitive interventions to improve the impact on food and nutrition security in South Africa were encouraging. Political will, limited delivery capacity, legislation restrictions and competing government priorities were listed as factors which would make policy combination difficult. Participants from the health, food and beverage industry and finance sector also mentioned that for combined policy approaches to work, research and investigation into these policies within a South African context would need to be done. Moreover, such policies would need to be undertaken with a strategic goal in mind and aligned with other government food and nutrition security policies.

All participants were strongly in favour of utilising the revenue generated from the tax for enhancing and capitalising on nutrition education campaigns. Studies have found that larger education or awareness campaigns would need to be introduced alongside the tax, together with supplementary efforts such as food subsidies or the provision of food vouchers. Education would also assist in consumer choice with regard to substituting SSBs with healthier alternatives [24].

Of note for future research regarding combined policy approaches and agriculture for nutrition policies, is the need for greater awareness and education about nutrition to improve general understanding and food choices; greater capacity for combined policy-approaches to operate within an obesogenic environment; focusing on the stability of household food supply as an important aspect of food security, and ensuring agriculture for nutrition policies in South Africa focus on minimising food wastage whilst improving the sustainability of crops in the current environmental climate.

### Sugar tax, negative externalities and education

Participants expressed concerns over the true motivation for the tax, in relation to the lack of ring-fencing of the funds for health-related initiatives such as health promotion. In terms of government spending in South Africa, it should be noted that the National Treasury allocates funds to each province based on equity. Thus, revenue generated is pooled into the general fiscus and then redistributed accordingly. Exceptions are funds ring-fenced for the Road Accident Fund and Universities South Africa [25]. Therefore, in general, South Africa has a policy against ring-fencing funds, however, greater allocations to the health budget could be made in response to this, as highlighted by the health sector participants. This needs to be combined with clearly outlined plans which illustrate where the additional funds will go in terms of combating the DBM. Specific attention should be placed on youth development and nutrition education programmes as mentioned by the participants.

Although participants were apprehensive about the ability of the tax to result in improved overweight and obesity outcomes at a ground level, they did note that the tax is still relatively new and that a longer timeframe would be required to assess the effects. These perceptions are consistent with a study that explored food taxes and their impact on competitiveness in the agri-food sector. The authors argued that changes in industry and consumer consumption patterns and subsequent health outcomes would take time to materialise and that some outcomes, such as the potential health effects, may require more time [26].

Participants were also concerned about the substitution effect, whereby consumers would likely switch to another beverage or food product that may not necessarily be healthier. The substitution effect in South Africa, in relation to fiscal policies such as the sugar tax, is largely unknown. Consumers may not substitute with a healthier alternative, unless larger education or awareness campaigns are introduced alongside the tax, together with supplementary efforts such as subsidising healthier food options or providing food vouchers [24]. Participants recognised these supplementary efforts as potential areas for ring-fencing the revenue generated by the tax on SSBs, in line with agriculture for nutrition interventions. Further research in a South African context is needed to understand consumer reaction in a local setting [27].

Future research on SSB tax could also look at the link between industry adaptation and health benefits. Participants attributed likely health benefits to industry adaptation of SSBs rather than reduced consumption. Industries have adapted their products by reducing the size of SSBs and reformulating by replacing sugar with alternative sweeteners [26].

This section highlights the overall perception that fiscal policies such as the tax on SSBs, should be implemented with side-along campaigns aimed at changing consumer behaviour. A combined policy approach is more likely to offset the negative externalities mentioned by participants, as well as create an opportunity for greater multi-disciplinary interaction and collaboration.

### Double burden of malnutrition

Participants perceived the DBM to be a concerning public health burden which places growing strain on economic growth, healthcare and social and youth development. Existing research highlights the global concern for the DBM in low-middle income countries [28]. Limited research is available on the overburdened public health system in South Africa; however, undernutrition and overnutrition are seen as public health crises globally [29–31].

A qualitative study on the attitudes and perceptions of urban South Africans in relation to the tax on SSBs highlighted similar perceptions to these study participants [32]. Both perceived the tax to be a solitary, income-generating mechanism used to enhance government funds rather than being focused on improving the DBM in South Africa.

An innovative suggestion from the consumer interest sector is having an independent, government-facilitated consumer movement group in South Africa. Research shows that civil society and media can contribute towards pressuring for ownership and accountability regarding malnutrition interventions. This includes following up on government commitments and ensuring that funds are well planned and accounted for [33]. This would give the consumer with lower socioeconomic status greater representation and voice when it comes to implementing fiscal policies such as the taxation on SSBs and any prospective combined policy. In future, when planning and implementing combined policy approaches, governments would do well to consult and work closely with communities and civil society organisations to ensure greater clarity, stakeholder commitment and support.

Overall, participants were generally in favour of combining the policies to improve the DBM. This would need to be a holistic approach which takes into consideration the need for education and market access of small-scale farmers as previously mentioned. Such approaches would allow for product distribution and behaviour change to take place, thus improving the ability of the nutrition-sensitive intervention to result in positive nutrition outcomes.

### Government function and scaling up nutrition-sensitive agricultural interventions

Participants frequently highlighted that before the tax on SSBs could be combined with a nutrition-sensitive agricultural policy, government would need to ensure that basic services are scaled up and run optimally. In light of the current government debt and mismanagement of government spending, the feasibility of combining these policies was though to be unlikely.

The need to create a demand for healthy foods within a market-based economy was mentioned as the next important step after ensuring there are effective basic services. Government, together with PPPs, needs to facilitate an effective value chain by ensuring proper infrastructure, services, awareness and education. Over and above this, government needs to ensure that small-scale farmers have market access and good product distribution in areas where populations need it the most. This would include distribution to street vendors and informal convenience stores or spaza shops.

A study by Gittelsohn, Laska, Karpyn, Klingler and Ayala [34] explored key areas to increase healthy food access in informal convenience stores. The main themes identified were, establishing relationships with stores and between stores and customers. Another study [35] investigated strengthening accountability systems to create healthy food environments to reduce global obesity. This study found quasi-regulatory approaches to be the most effective in combating industry opposition and government reluctance. Agriculture for nutrition policies can combine these insights into future interventions, to effectively function in a market-based economy.

Most participants were in favour of scaling up nutrition-sensitive agricultural interventions and mentioned that government can focus on not only agriculture but the consumer. The participants agreed that there is a need for greater policy coherence and stakeholder involvement to improve implementation, monitoring and evaluation. This is consistent with research which identified poor coherence among South Africa’s numerous food and agricultural policies [36]. Incoherence was related to unclear or conflicting goals and objectives of the policies and lack of accountability of responsible parties [36].

A policy review by Hendriks and Olivier [37] found that South African agri-food policies require greater coherence between policies, ‘power players’ and independent actors – especially when making, implementing, monitoring and evaluating agri-food programmes [36, 37]. The United Nations Standing Committee on Nutrition (UNSCN) identified similar challenges which impede the success of nutrition-sensitive agriculture for nutrition policies [38].

Past research has found mixed and inconclusive evidence on agriculture for nutrition interventions, due to the use of multifaceted nutritional indicators. Recommendations were made for governments to focus on identifying and agreeing on key indicators to more effectively measure the impact of these interventions [6, 39]. Thus, the composite indicators identified by participants can be used in the monitoring and evaluation of agriculture for nutrition interventions in future. The indicators assess the impact of these policies on health and nutrition across the value chain. The indicators mentioned by participants are production indicators, purchasing patterns, consumption and dietary diversity patterns and health status indicators.

### Collaboration and enabling environments

For government to scale up nutrition-sensitive agricultural policies and combined policy approaches, government needs to create an enabling environment for intersectoral collaboration, PPP and community and stakeholder engagement. Improving intersectoral collaboration and PPP is one of the pillars detailed in the National Policy on Food and Nutrition Security [18]. Currently, the coordination mechanisms to align the response and goals of various sectors and government departments remains underdeveloped and unbudgeted for.

An enabling environment and enhanced collaboration will also ensure a greater commitment towards a Health in All Policies approach whereby the improvement of health is incorporated into collaborative decision-making across sectors and policies [40].

The recurring notion of nutrition champions was highlighted as being essential for creating enabling environments. Ample literature supports the notion of nutrition champions. According to the Scaling Up Nutrition movement, a nutrition champion raises awareness about nutrition and assist in placing nutrition-related issues on the political agenda [41, 42]. Different levels of nutrition champions exist, from high-level to working-level and grassroots champions such as teachers and community leaders [41, 42]. For nutrition champions to be effective, they need to be well-connected and trusted within informal and formal social networks [43].

Research shows that nutrition champions can transfer information, resolve conflicts and positively change perceptions in favour of a nutrition agenda. This is linked to their extensive knowledge of and experience in nutrition and their ability to develop relationships with different stakeholders [44]. The identification of potential champions through stakeholder mapping is important as it ensures the selection of context-appropriate champions. Champions should be aware of their roles and responsibilities and be provided with support to ensure long-term commitment to assigned policies and interventions [43, 44]. Therefore, nutrition champions would be crucial in the development of combined policy approaches, especially in driving the alignment of nutrition-sensitive and nutrition-specific policies and programmes. In particular, nutrition champions could assist in driving such combined policy approaches linked to the taxation on SSBs in South Africa.

### Economic environment

Participants believed that combining nutrition-sensitive and nutrition-specific policies would assist with job creation and improve youth development and overall health and productivity. However, this would need serious government commitment to be effective. Government would need to consider other competing priorities, and in the case of ring-fencing funds for agricultural interventions, would need to consider factors such as education, awareness and opposing market forces.

A recent study conducted in Mexico which researched employment changes associated with the tax on SSBs found no significant changes in employment [44]. More research in a South African context will be required to assess the impact of the sugar tax on the broader economy. No literature is available on potentially combining the tax on SSBs with a policy aimed at nutrition-sensitive agricultural interventions as a means to prevent job losses and improve food and nutrition security. This research underlines the potential for combining the current tax on SSBs with an agriculture for nutrition policy to offset the potential negative externalities which may arise from the tax. Whilst allowing for benefits across the value-chain, from the small-scale farmer to the consumer, when combined with mass education campaigns and monitoring and evaluation.

Many feasibility issues to combined policy approaches in South Africa were voiced by the participants. Ongoing research around such approaches in other developing and developed countries would provide further insight into the barriers and feasibility of combining nutrition-sensitive and nutrition-specific policies in the future, especially with regards to the tax on SSBs and agriculture for nutrition policies.

## Conclusion

Overall, perceptions with regard to combining the taxation on SSBs with nutrition-sensitive agricultural policies to improve overall health and nutrition in South Africa were positive. However, the participants questioned the relative feasibility of combining these nutrition-sensitive and nutrition-specific policies in the current socioeconomic and political environment. Political will, limited delivery capacity, legislation restrictions and competing government priorities were listed as factors which would make policy combination difficult. Combining these policies was seen as a positive way of overcoming the alleged collateral damage linked to the tax, with specific focus on developing small-scale farmers in a South African context.

Combining these policies would allow for enhanced focus on developing small-scale farmers, creating jobs and ensuring adequate household food security in rural populations. Participants perceived that combining nutrition-sensitive and nutrition-specific policies would have a positive impact on food and nutrition security, the DBM and the South African economy. However, it would need serious government commitments for it to be effective, including considering other competing priorities. In the case of ring-fencing funds for agricultural interventions, government would need to consider factors such as education, awareness and opposing market forces.

Participants consistently referred to education as an essential element required to successfully change behaviour and ensure a positive and sustainable consumer impact of the taxation on sugar and other combined policy approaches.

## Recommendations

More research and investigation into these combined policy approaches in a South African context is required. Prospective research and impact analyses should also be done in order to assess the impact that sugar tax has on jobs, funds allocated to the health section and price elasticities. Furthermore, updated health and nutrition surveys, together with more research on the weighting of dietary causes of obesity against other causes in South Africa would allow for improved targeting of future health-related interventions.

In order to implement and scale up agriculture for nutrition policies, the South African government would need to ensure that basic services are run optimally. In addition, improved intersectoral collaboration with PPPs, non-governmental organisations and civil society is needed to create a demand for healthy goods in a market-based economy. Greater intersectoral collaboration would also allow for greater buy-in from key stakeholders and ensure greater accountability, monitoring and evaluation of programme outcomes.

There is a need for greater policy coherence and stakeholder involvement to improve implementation, monitoring and evaluation of interventions in South Africa. Focus should be placed on greater coordination mechanisms, to ensure minimal overlap and animosity between and within departments. This will allow for greater responsibility and accountability of various stakeholders and a greater platform from which to scale up successful agriculture for nutrition policies.

### Study limitations

The study frame was restricted to the particular sectors in Gauteng, South Africa because of technical and logistical convenience, and therefore may not be representative of the perceptions of other sectors and/or other provinces of South Africa.

The small sample population limits the generalisation of the study results; however, participants were purposively sampled and the sample size was determined through data saturation as is largely recommended in qualitative research [45].

Another limitation is possible sensitivity bias from the participants from the various sectors, due to the political and economic sensitivity surrounding the Health Promotions Levy at the time of the interviews.

## Supplementary information

**Additional file 1.** Interview Guide: In-depth interview guide used by principal researcher to guide the interviews and in-depth discussions during the data collection phase.

**Additional file 2.** Spider diagram figures: The network function on ATLAS.ti 8 was used to recreate spider diagrams on the main themes and look for further patterns, connections and relationships between and within themes. Spider diagrams are labelled: Food and Nutrition Security, Government Function, Negative externalities linked to the sugar tax, Nutrition-agricultural policies, Ring-fencing funds to offset the negative externalities and Sugar Tax.

## Data Availability

The datasets generated and/or analysed during the current study are not publicly available due [to protect individual autonomy and confidentiality] but are available from the corresponding author on reasonable request.

## Abbreviations

BMI: Body Mass Index
DBM: Double burden of malnutrition
NCDs: Non-communicable diseases
NGO: Non-governmental organisation
PPP: Public–private partnership
SADHS: South Africa Demographic and Health Survey
SSBs: Sugar-sweetened beverages
Stats SA: Statistics South Africa
UNSCN: United Nations Standing Committee on Nutrition
USA: United States of America
WHO: World Health Organization
WWF: World Wide Fund for Nature

## Footnotes

1 They aim to tackle food insecurity, undernutrition and overweight and obesity concurrently [10].
