# Supplementary material for: A qualitative analysis of perceptions of various stakeholders on nutrition-sensitive agricultural interventions, including the taxation on sugar-sweetened beverages (SSBs), to improve overall health and nutrition in South Africa
Source: BMC Public Health. 2020 Sep 3;20:1342. doi: 10.1186/s12889-020-09440-8 (PMC7469266; doi:10.1186/s12889-020-09440-8)
Supplement: Supplementary file 1 — Additional file 1. Interview Guide: In-depth interview guide used by principal researcher to guide the interviews and in-depth discussions during the data collection phase. [file 12889_2020_9440_MOESM1_ESM.docx]

**IN-DEPTH INTERVIEW GUIDE**

Good morning/afternoon Mr/Mrs (Surname). Thank you for taking the time to meet with me today, I really appreciate it. I would like to start by formally introducing myself and briefly explaining the purpose of my **research project** and what you **can expect** from today’s interview.

I am Tayla Kaltenbrun a Masters Student at the University of Stellenbosch, I am conducting research to gain a better understanding of the perceptions of various stakeholders on ‘potentially’ combining nutrition-sensitive agricultural interventions (interventions which are pro-nutrition), such as the biofortification of crops, with the taxation on Sugar Sweetened Beverages (fiscal policy introduced in April 2018 in South Africa) to **improve overall health and nutrition** in South Africa.

It is important to understand whether various stakeholders believe this suggestion to be **feasible** and to recommend other ways in which ‘nutrition-sensitive agricultural interventions’ can be scaled-up. This information is necessary for implementing future policies and for assisting the South African government to combat overnutrition and undernutrition during the current economic climate.

The reason I am interviewing you today is because you are a subject matter expert within your sector, and I believe that I can learn a lot from both your knowledge and experience.

Today’s interview will not take more than 45-60 minutes of your time. I would prefer if today’s interview could be very open, conversational and informed. As you are the professional, I would like to learn as much as possible from you. The interview is, and will remain, completely **confidential and anonymous** – your name and company will not be identified in the research report. As such I will be referring to you as ‘Identifier’ during the interview.

I would like to now ask for your permission to audio-record our conversation; I will be using both my phone and my iPad as recording devices. Before we begin, I would like to ask if you could please read through and sign the informed consent form, including the section on consenting to being audio-recorded.

Do you have any questions with regards to today’s interview before I proceed?

[Terminology: nutrition-sensitive policies have an **indirect** impact on nutritional status, these policies typically involve other sectors such as agriculture, to address the underlying causes of malnutrition. Nutrition-specific policies have a **direct** impact on the prevention and treatment of malnutrition, such as fiscal tax policies.]

For the purpose of today’s interview, I will be shortening sugar-sweetened beverages to SSBs.

**CONTACT DETAILS:**

PRINCIPAL INVESTIGATOR: Tayla Kaltenbrun

CONTACT NUMBER: 072 506 7128

[21878188@sun.ac.za](mailto:21878188@sun.ac.za)

SUPERVISOR: Prof Lisanne Du Plessis

CONTACT NUMBER: 012 938 9175

lmdup@sun.ac.za

…………………………………………………………………………………………………

**1. Demographics**

- 1. Gender – Male / Female (Circle and say gender in audio-recording)
  2. What is your date of birth?
  3. What is your highest level of education?
  4. What is your general job title?
  5. How many years of experience do you have in your relevant field / sector?
  6. Could you please talk me through what your role is in your sector?

…………………………………………………………………………………………………..

**2. Understanding of topic area**

2.1 What is your current perception on the state of South Africa’s (SAs) food and nutrition?

2.2 What is your understanding of South Africa’s tax on SSBs? / What is your professional opinion on South Africa’s tax on SSBs?

*[Based on response, may need to explain SAs tax on SSBs - SSBs are defined as beverages which contain nutritive sweetners such as: high-fructose corn syrup (HFCS), sucrose or fruit-juice concentrate. The policy excludes unsweetened milk and 100% fruit-juice in the definition.Tax will be set at a tax rate of 2.29 cents per gram of sugar. This will affect the majority of the popular SSBs in South Afica.]*

2.3 Do you perceive that there will be any health benefits gained from the tax on SSBs?

2.3.1 If yes, how does or will it improve health?

2.3.2 If no, why do you say that it will have no perceived health benefits?

*The government has various policies that link agriculture and nutrition (nutrition-sensitive), to ensure that agricultural practices in South Africa are pro-nutrition i.e. that they achieve health and nutrition goals such as reducing malnutrition.*

2.4 Are you aware of any government policies aimed at nutrition, that have an agricultural component?

2.4.1 If yes, could you please elaborate on these and their impact in your

sector?

2.4.2 If no, do you believe that we should have nutrition-sensitive agricultural policies and interventions in South Africa, and why?

OBJECTIVE 1: What are the perceptions with regards to; combining nutrition-specific and nutrition-sensitive interventions to improve the impact on food and nutrition security in South Africa?

**3. Food and Nutrition Security**

*Food security is defined as the availability and accessibility to affordable, adequate, safe, nutritious and culturally appropriate foods in order to maintain health and lifestyle.*

3.1 In your professional opinion, do you think the government (and private companies) do enough to ensure food and nutrition security in South Africa?

3.2 Do you think South Africa’s tax on SSBs will have any impact on nutrition outcomes, specifically relating to improving food security?

3.2.1 If yes, what impact do you foresee the tax having?

3.2.2 If no, why do you believe there won’t be any impact?

*Agricultural policies which are pro-nutrition, integrate the health and agricultural sector to jointly tackle key challenges such as food insecurity and malnutrition.*

3.3 If government could combine the tax on SSBs with policies aimed at agriculture (i.e. utilising revenue generated), would this lead to further improvements in nutrition outcomes and food security?

3.3.1 Is this something that you have or can actively considered within your

sector?

3.2.3.1 If yes, why has/could this been/be considered in your sector?

3.2.3.2 If no, why have/would you not considered this in your sector?

3.4 Would there be any long-term benefits or consequences, for South Africa in general and in your sector, if these two policies were to be combined?

3.4.1 What would the long-term benefits or consequences be on nutrition

and food security?

3.4.2 What could be done to combat the negative consequences?

3.4.3. What could be done to ensure that positive benefits are prolonged?

3.5 Are you currently engaging with any other sectors to improve overall health and/or nutrition in South Africa?

3.5.1 If yes, how are you engaging with other sectors to

improve health?

3.5.2 If no, why are you currently not engaging with other sectors to improve

health?

*The National Treasury policy paper on the tax on SSBs doesn’t specify where the revenue generated will be allocated. By potentially combining this policy paper with a sub-section on agriculture, government could direct revenue towards pro-nutrition agricultural policies.*

3.6 Should the government allocate the revenue generated to incentivise small-scale and/ or sugar-cane farms to designate a portion of their land to growing fruit and vegetable crops (or other nutritious crops i.e. biofortified crops)?

3.6.1 If yes, what incentives would be the most effective in achieving this?

3.6.2 If no, why is this not a feasible suggestion?

3.6.3 Could the government utilise the revenue generated to subsidise healthier food options?

3.7 Where would you suggest the revenue generated should go in order to improve nutrition and food security?

3.8 How could the government ensure that an effective value chain is in place, so that the nutritious foods grown will being easily available and accessible to community members (population most at need)?

3.9 How could they link agriculture for nutrition and the policy paper on the taxation of sugar-sweetened beverages to improve food security, mainly in terms of access?

OBJECTIVE 2: What are the perceptions with regards to; combining nutrition-specific and nutrition-sensitive interventions to improve the impact on the double burden of malnutrition in South Africa?

**4. Double Burden of Malnutrition**

*The double burden of malnutrition refers to having overnutrition and undernutrition within the same household/ community.*

4.1 How are overnutrition and undernutrition public health concerns in South Africa?

4.2 In your professional opinion, which one poses a greater public health concern and why?

4.3 Does your sector directly or indirectly impact the double burden of malnutrition?

4.3.1 In what way do they impact the double burden of malnutrition?

4.4 Has your sector done anything to improve overnutrition and/ or undernutrition and what have they done?

4.4.1 If nothing has been done, why has nothing been done?

4.5 How do nutrition-sensitive agricultural policies / interventions influence overnutrition and undernutrition?

4.6 I asked earlier if you believe the tax on SSBs will lead to improved health in South Africa – in what way will it contribute to improving overnutrition specifically?

4.6.1 Will it have any impact on undernutrition in South Africa?

4.7 If the tax on SSBs is combined with nutrition-sensitive agricultural policies / interventions, would it result in improved overnutrition and undernutrition outcomes?

4.7.1 In what way, do you perceive the combination of the policies will impact

undernutrition specifically?

OBJECTIVE 3: What are the perceptions with regards to; combining nutrition-specific and nutrition-sensitive interventions to improve the impact on the economic environment in South Africa?

**5. Economic Environment**

5.1 How effective do you perceive fiscal policies to be on reducing the purchasing and consumption the taxed item?

5.1.1 Pertaining to the new National Treasury policy paper on the tax on SSB - how effective do you perceive this fiscal policy will be on reducing the purchasing and consumption of SSBs?

5.2 Would financial incentives, provided by the government, be enough to enforce combining nutrition and agricultural polices?

5.2.1 What else could the government do to enforce combining nutrition

and agricultural policies (in your sector)?

5.2.2 Would this in any way impact your sector?

5.3 Would linking direct and indirect nutrition policies have a positive impact on South Africa’s economy? In what way?

5.4 From your sectors opinion, can the tax on SSBs be considered regressive in nature i.e. placing a larger economic strain on lower socio-economic groups?

OBJECTIVE 4: To determine how South African governance can create enabling environments to combine nutrition-specific and nutrition-sensitive interventions.

**6. Enabling Environments**

*An environment which facilitates smooth inter-sectoral collaboration.*

6.1 What lessons can be learnt from past inter-sectoral collaboration? Example of inter-sectoral collaboration *department of health and department of education – school feeding schemes*

6.2 How can the government improve collaboration between sectors, so that nutrition and food security goals are aligned and met?

6.3 What else could the government do to ensure that these combined policies, move from policy to action on a ground level?

6.4 What framework could the government put in place to ensure that the revenue generated from the tax on SSBs is directed towards nutrition-sensitive agricultural intervention?

OBJECTIVE 5: To determine how South African governance can scale-up on agriculture for nutrition policies.

**7. Scaling up on nutrition-sensitive agricultural policies**

*In order for government to scale-up on agriculture for nutrition policies, appropriate indicators need to be selected to monitor and evaluate their effectiveness, efficiency and impact on health and nutrition in South Africa.*

7.1 What indicators could the government use to monitor and evaluate nutrition-sensitive agriculture policies for effectiveness/ efficiency/ impact on health and nutrition?

7.2 Should the South African governance scale-up on agriculture for nutrition policies / interventions?

7.2.1 How would they scale-up such policies and interventions – from pilot to public scale?

7.3 What are the policy weaknesses exist in South Africa?

7.3.1 How would these policy weaknesses impact combining nutrition-sensitive and nutrition-specific policies?

7.3.2 What can be done differently to ensure these weaknesses are overcome?

7.4 What are the operational weaknesses that exist in South Africa when converting policy to ground-level interventions?

7.4.1 What should be done differently in the future in order to successfully scaling up nutrition-sensitive agricultural policies / interventions?

**8. Recommendations**

8.1 Is there anything else that you would like to add to our discussion today?

8.2 Do you have any recommendations with regards to research this area?

*Additional probes to be used when needed:*

1. *What do you mean by that?*
2. *Could you please give me an example?*
3. *Please could you explain that a little bit more?*
4. *Why do you say that?*
5. *When?*
6. *How?*

**CONCLUSION**

That brings me to the end of our questions. Thank you once again Mr/Mrs (Surname) for taking the time to speak with me today, your insight is truly appreciated. How did you find the interview? Should I require some clarity on anything that we have covered today, would it be okay if I contacted you regarding this? Please take this as a small token of appreciation for your time.
