# Supplementary material for: A qualitative analysis of perceptions of various stakeholders on nutrition-sensitive agricultural interventions, including the taxation on sugar-sweetened beverages (SSBs), to improve overall health and nutrition in South Africa
Source: BMC Public Health. 2020 Sep 3;20:1342. doi: 10.1186/s12889-020-09440-8 (PMC7469266; doi:10.1186/s12889-020-09440-8)
Supplement: Supplementary file 2 — Additional file 2. Spider diagram figures: The network function on ATLAS.ti 8 was used to recreate spider diagrams on the main themes and look for further patterns, connections and relationships between and within themes. Spider diagrams are labelled: Food and Nutrition Security, Government Function, Negative externalities linked to the sugar tax, Nutrition-agricultural policies, Ring-fencing funds to offset the negative externalities and Sugar Tax. [file 12889_2020_9440_MOESM2_ESM.pdf]

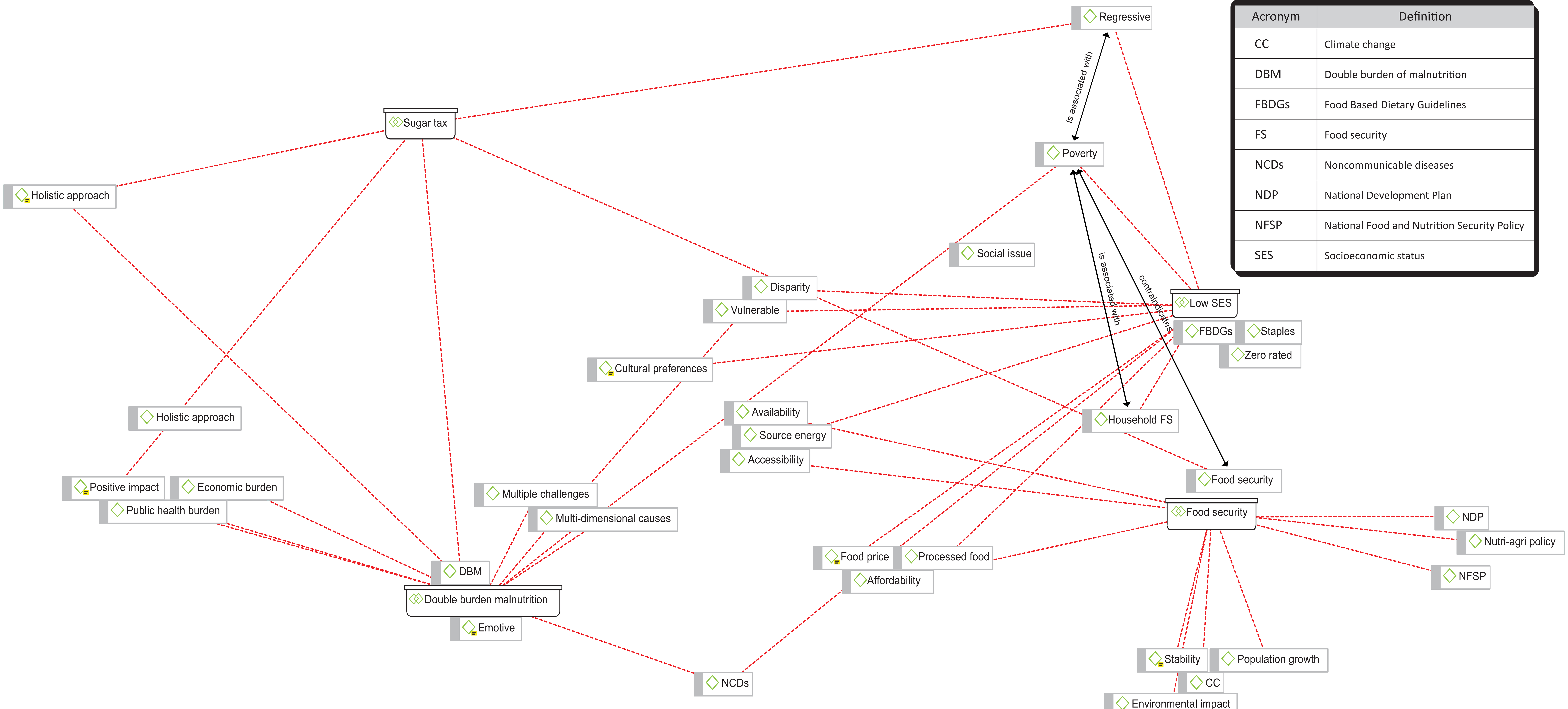

Acronym	Definition
CC	Climate change
DBM	Double burden of malnutrition
FBDGs	Food Based Dietary Guidelines
FS	Food security
NCDs	Noncommunicable diseases
NDP	National Development Plan
NFSP	National Food and Nutrition Security Policy
SES	Socioeconomic status

Figure 1: Food and Nutrition Security

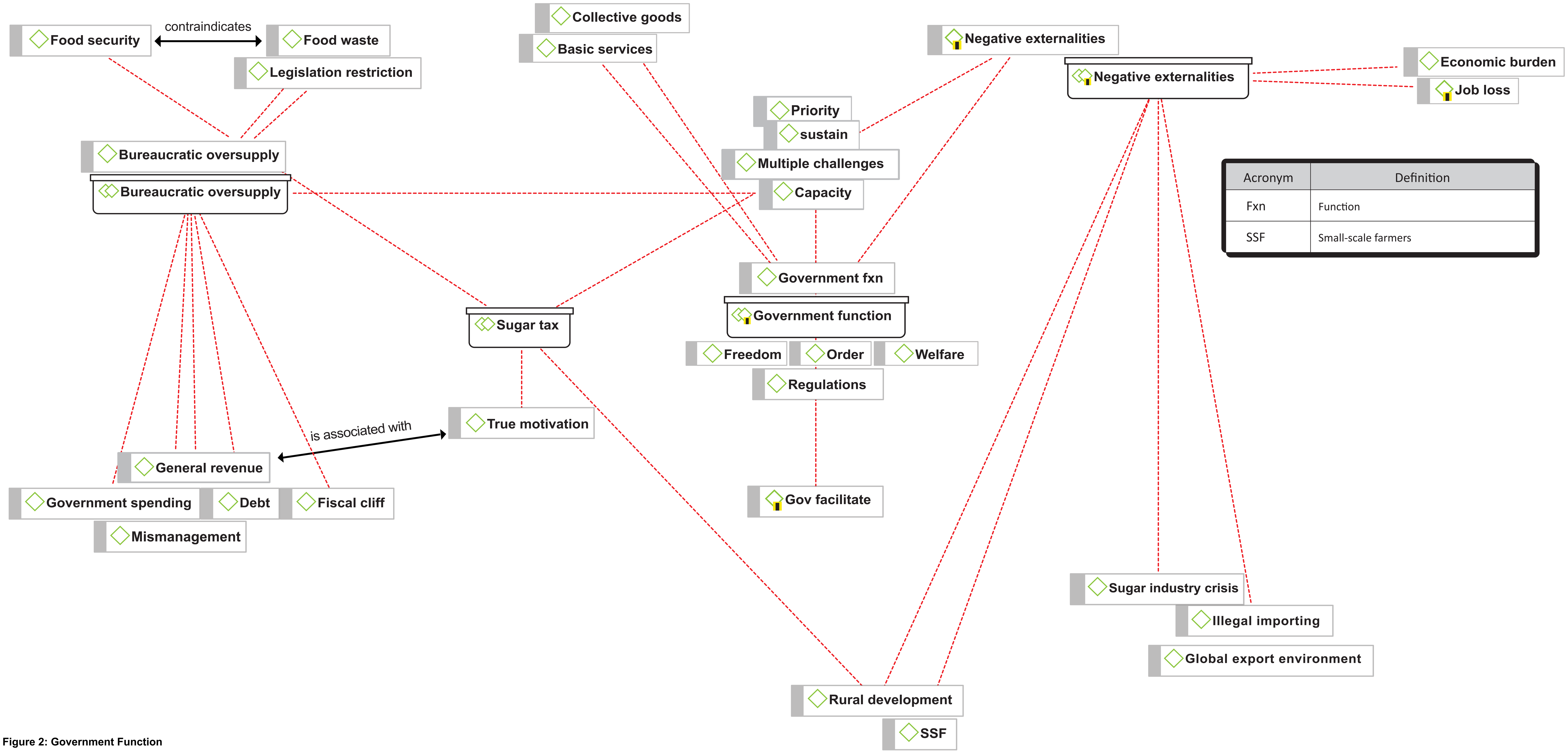

Acronym	Definition
Fxn	Function
SSF	Small-scale farmers

Figure 2: Government Function

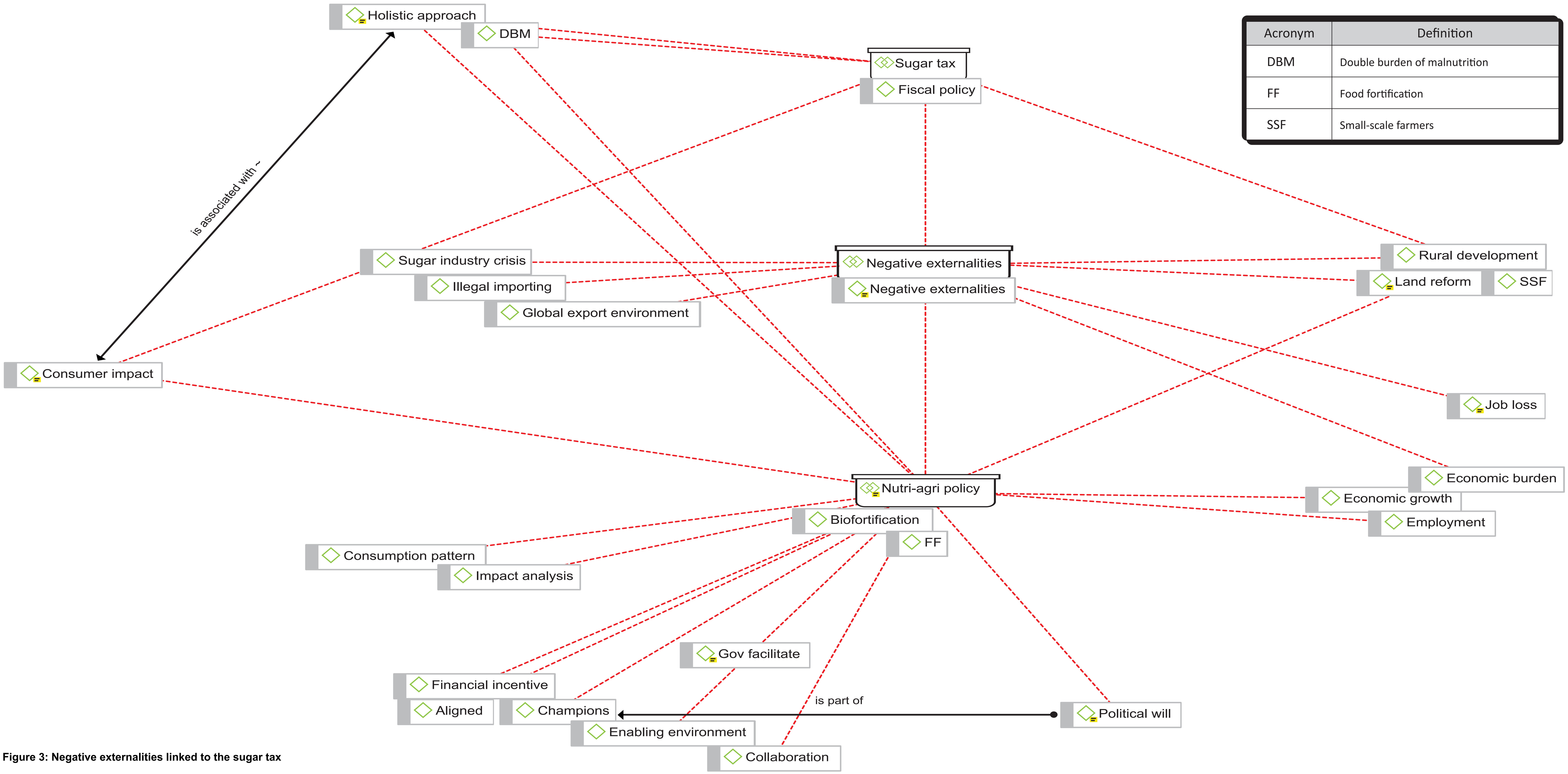

Acronym	Definition
DBM	Double burden of malnutrition
FF	Food fortification
SSF	Small-scale farmers

Figure 3: Negative externalities linked to the sugar tax

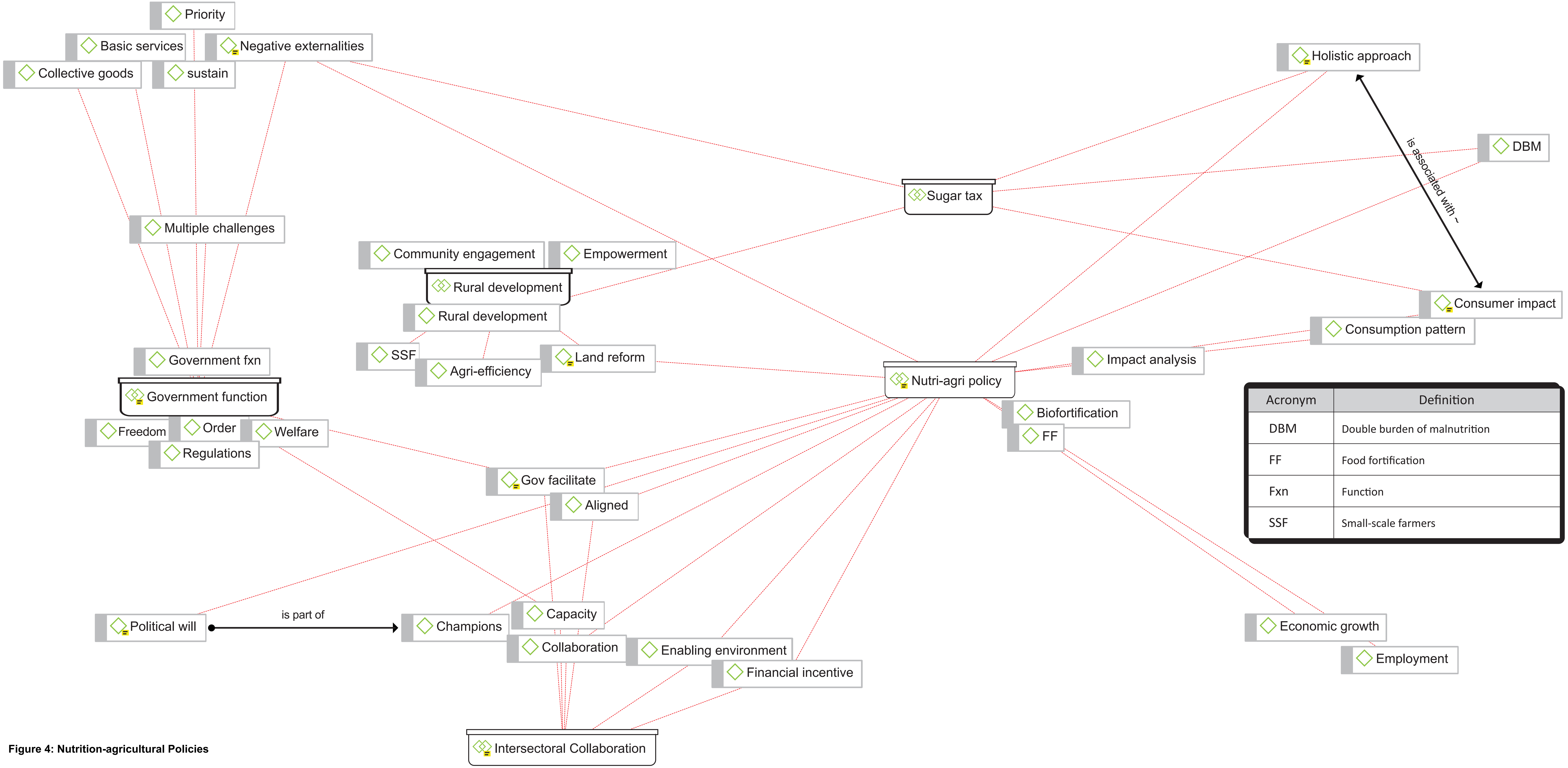

Acronym	Definition
DBM	Double burden of malnutrition
FF	Food fortification
Fxn	Function
SSF	Small-scale farmers

Figure 4: Nutrition-agricultural Policies

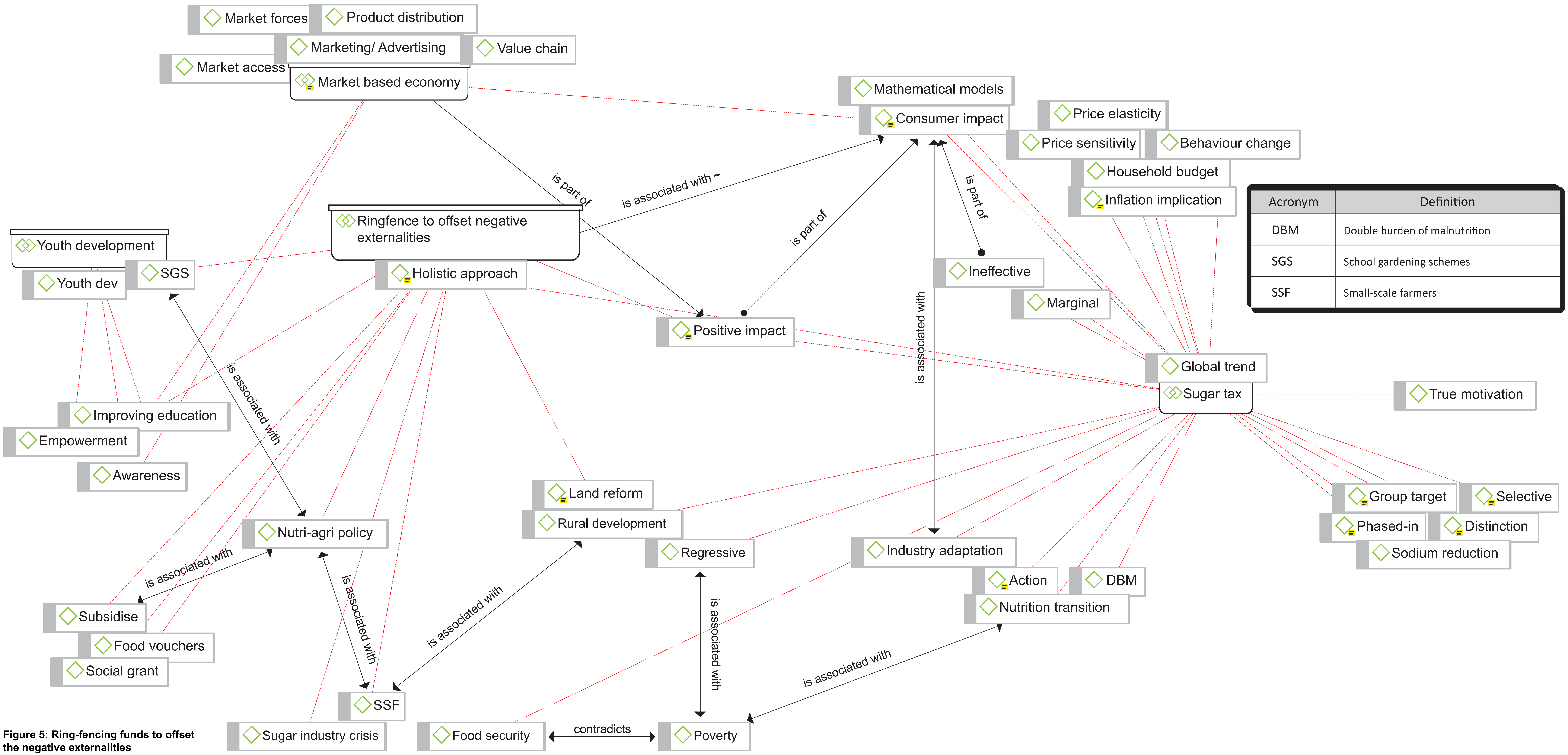

Acronym	Definition
DBM	Double burden of malnutrition
SGS	School gardening schemes
SSF	Small-scale farmers

Figure 5: Ring-fencing funds to offset the negative externalities

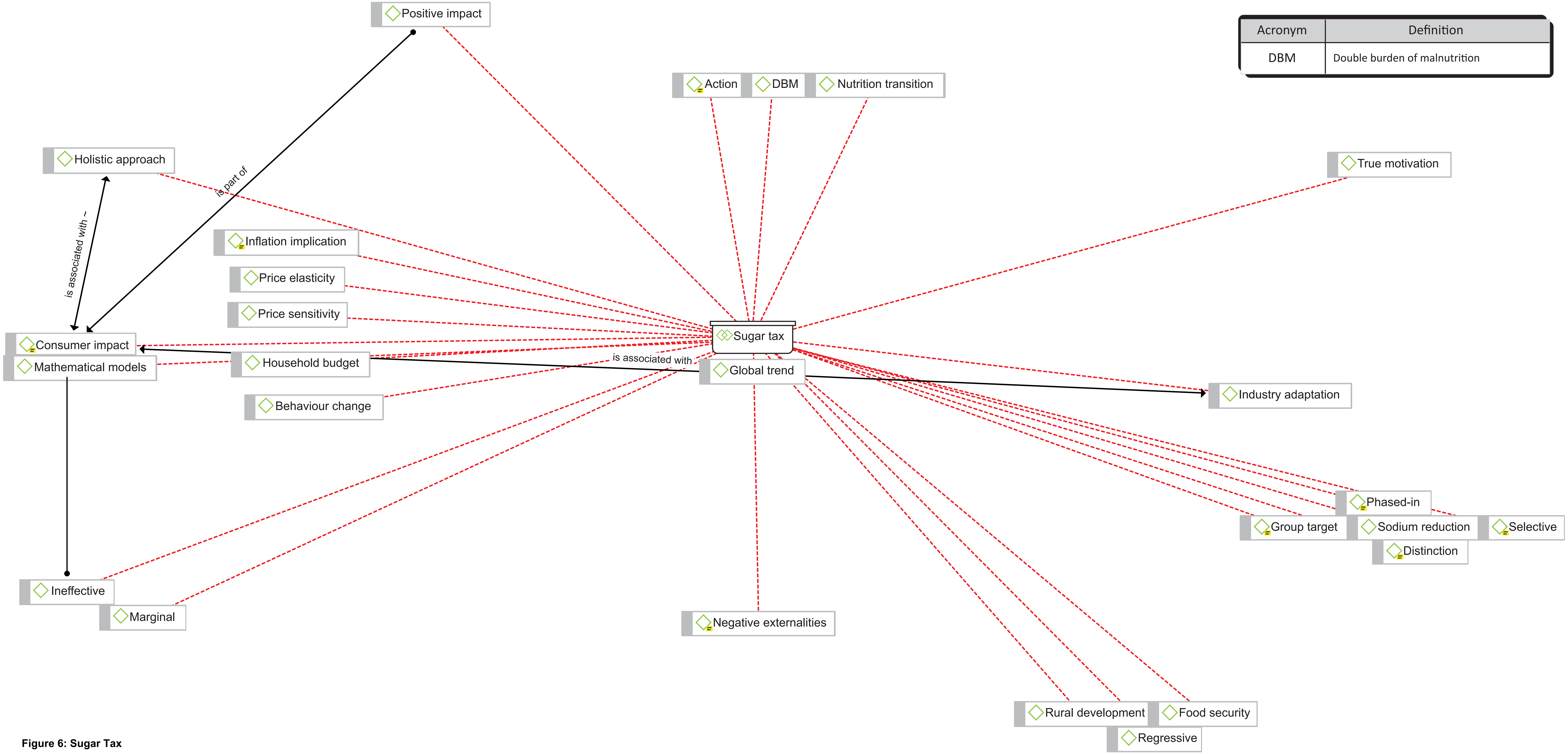

Acronym	Definition
DBM	Double burden of malnutrition

Figure 6: Sugar Tax
